# SUMOylation modification-mediated cell death

**DOI:** 10.1098/rsob.210050

**Published:** 2021-07-14

**Authors:** Zenghua Sheng, Jing Zhu, Ya-nan Deng, Shan Gao, Shufang Liang

**Affiliations:** State Key Laboratory of Biotherapy and Cancer Center, West China Hospital, Sichuan University, and Collaborative Innovation Center for Biotherapy, No.17, 3rd Section of People's South Road, Chengdu, 610041, People's Republic of China

**Keywords:** SUMOylation, cell death, ferroptosis, GPX4

## Abstract

SUMOylation dynamically conjugates SUMO molecules to the lysine residue of a substrate protein, which depends on the physiological state of the cell and the attached SUMO isoforms. A prominent role of SUMOylation in molecular pathways is to govern the cellular death process. Herein, we summarize the association between SUMOylation modification events and four types of cellular death processes: apoptosis, autophagy, senescence and pyroptosis. SUMOylation positively or negatively regulates a certain cellular death pattern depending on specific conditions including the attached SUMO isoforms, disease types, substrate proteins and cell context. Moreover, we also discuss the possible role of SUMOylation in ferroptosis and propose a potential role of the SUMOylated GPX4 in the regulation of ferroptosis. Mapping the exact SUMOylation network with cellular death contributes to develop novel SUMOylation-targeting disease therapeutic strategies.

## Graphical abstract

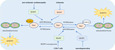

## 1. Introduction

Small ubiquitin-like modifier (SUMO) is a highly conserved molecule to conjugate to the lysine (Lys) residue of a substrate protein in the post-translational modification (PTM) process [1,2]. Five SUMO isoforms, named SUMO1, 2, 3, 4 and 5, are present in mammalian cells [2]. SUMO2 and SUMO3 have 96% homology [2]. SUMO4 and SUMO5 are the least studied SUMO forms to date. SUMO4 is encoded by the human TAB2 gene, which is similar to SUMO2/3 except that the SUMO4 sequence contains a proline 90 instead of a glutamine [3]. SUMO5, a newly discovered SUMO family member, is highly expressed in several primate tissues such as testes and peripheral haemolymph [4].

The SUMO molecule undergoes a similar enzymatic reaction cascade to ubiquitin to conjugate to a target protein, involving the sequential action of E1, E2 and E3 enzymes [1–4]. Conversely, the deSUMOylation and precursor processing are catalysed by the SUMO-specific proteases called SENPs, including SENP1, SENP2, SENP3, SENP5, SENP6 and SENP7 in humans (table 1) [5,6]. SENPs are broadly divided into three categories according to their different subcellular localizations and substrates. The first category is SENP1 and SENP2, which are concentrated in nuclear pore or the PML nuclear bodies in interphase cells. SENP1 and SENP2 have broad isopeptidase activities for SUMO1, SUMO2 and SUMO3. However, SENP2 prefers SUMO2 over SUMO1 and SUMO3 as the substrate, whereas SENP1 is most active on SUMO1, followed by SUMO2 and SUMO3. The second category includes SENP3 and SENP5, and they prefer to enrich in the nucleolus. SENP3 and SENP5 have a high specificity for SUMO2/3 and deconjugate SUMO1 from the substrate with a low efficiency. The third category, SENP6 and SENP7, are mainly found in the nucleoplasm. SENP6 and SENP7 have a pronounced preference for SUMO2/3 cleavage, but they most efficiently cleave di- and poly-SUMO2/3 chains. SENP6 and SENP7 contribute little to precursor processing, whereas SENP1, SENP2 and SENP5 participate in SUMO maturation [6]. The restrictive SUMO precursor and the mature SUMO subtype, as well as the SENP family, together determine the balance of cellular SUMO modification.

**Table 1.** The subcellular localization and biological function of SENPs.

name	localization	substrate preference	function		refs
			deSUMOylation	precursor processing	
SENP1	nuclear pore and PML nuclear bodies	SUMO1 > SUMO2, SUMO3	√	√	[5,6]
SENP2	nuclear pore and PML nuclear bodies	SUMO2 > SUMO1, SUMO3	√	√	[5,6]
SENP3	nucleolus	SUMO2/3	√	unknown	[5,6]
SENP5	nucleolus	SUMO2/3	√	√	[5,6]
SENP6	nucleoplasm	poly-SUMO2/3	√	×	[5,6]
SENP7	nucleoplasm	poly-SUMO2/3	√	×	[5,6]

A large number of proteins have been identified to covalently link with SUMO, which results in changes in their fate, stability and function. Recent data suggest that more than 6000 proteins are modified by SUMO to date, and most of object belongs to nuclear protein [7]. Moreover, SUMOylation acts an important role in various cellular processes of several types of cell death [8]. In this review, we mainly focus on the function of SUMOylation in four types of cell death: apoptosis, autophagy, senescence and pyroptosis. In addition, we also highlight the potential relations between SUMOylation and ferroptosis, and propose a potential role of the SUMOylated GPX4 in ferroptosis, which becomes a research hotspot of SUMOylation-mediated cell death.

## 2. SUMOylation-mediated cell death

Multiple studies have shown that SUMOylation is involved in cellular death processes including apoptosis, autophagy, senescence and pyroptosis. An understanding of the molecular mechanism of SUMOylation-mediated cell death can contribute to development of drug target and cancer treatment strategies.

### 2.1. SUMOylation-mediated apoptosis

Apoptosis is a caspase-mediated programmed cell death, which mainly includes mitochondrial pathway, endoplasmic reticulum pathway and death receptor pathway according to the different initiation stages of apoptosis. Associations between SUMOylation and apoptosis have been extensively studied. For example, the activation of apoptosis mediated by SUMO modification is one of the important mechanisms of cardiovascular disease [9,10]. An abnormal increase of a de-SUMOylation enzyme is related to non-ischaemic cardiomyopathy. The overexpression of SENP5 in murine hearts causes a significant reduction of global SUMO2/3 conjugation and increases cardiomyocyte death via mitochondrial apoptosis pathway, ultimately leading to cardiomyopathy [9]. A mechanistic model showed that SENP5 upregulation in cardiomyocytes downregulates SUMO2/3 conjugation to the fission GTPase dynamin-related protein 1 (Drp1), which is accompanied by enlargement of mitochondria and elevated cell death [9]. In addition, the knockdown of SUMO1 level leads to premature death and cardiovascular disease in murine models [10]. It is consistent that the SUMO1 mutant mice are rescued successfully by re-expressed SUMO1 in cardiomyocytes, which indicates an essential threshold level of SUMO1 for normal cardiac structural development [10]. Taken together, the reduction of global SUMOylation level is one of the important mechanisms that induce apoptosis of cardiomyocytes, ultimately leading to cardiomyopathy.

**Figure 1.**
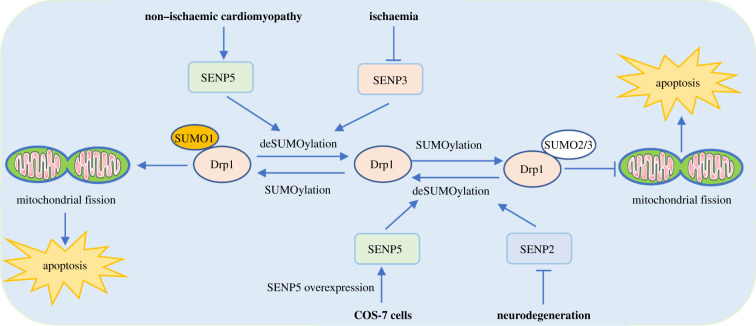
SUMO1 or SUMO2/3 can conjugate with a same target protein Drp1, which leads to a positive and negative regulation of cell apoptosis. The SUMO1-conjugated Drp1 significantly accelerates mitochondrial fission, ultimately facilitating the apoptosis process. Conversely, the SUMO2/3-conjugated Drp1 effectively delays mitochondrial fission and inhibits cell apoptosis.

Notably, the same substrate protein will perform specific roles by conjugation with SUMO1 or SUMO2/3. For instance, modifications of Drp1 by SUMO1 and by SUMO2/3 have different functional consequences to mitochondrial apoptosis activity (figure 1). Drp1 belongs to a member of the dynamin family of GTPases, and it is composed of GTP-binding, middle, insert B and C-terminal GTPase effector domains. In response to apoptotic stimuli, Drp1 is recruited into the outer mitochondrial membrane to create a collar-like fission apparatus by multimerizations, which leads to scission and divides mitochondria, finally promoting cell apoptosis [11]. Drp1 is a target of SUMOylation in an apoptotic event [9,12–18]. Drp1 is modified by all three conjugable SUMO isoforms (SUMO1, SUMO2/3) within the B domain by preferentially modifying at multiple non-consensus sites, including K532, K535, K558, K568, K594, K597, K606 and K608 [12]. In COS-7 cells, the overexpression of SUMO1 results in mitochondrial fragmentation and the stabilization of Drp1 protein [13], whereas transient transfection of SENP5 could rescue the SUMO1-induced mitochondrial fragmentation and catalyses the cleavage of SUMO1 from Drp1, which causes to decelerate mitochondrial fission [14]. MAPL is the first mitochondrial-anchored SUMO E3 ligase, and it also regulates mitochondrial fission via enhancing the binding between SUMO1 and Drp1 [15]. In neurodegenerative diseases, the SUMO1 modification of endogenous Drp1 is eliminated by SENP2 not SENP5 [16]. The overexpression of SUMO2 can constitute a cytoprotective pathway to induce cell apoptosis for severe ischaemia [17]. But another report from a model of ischaemia shows that SUMO2/3 conjugations are massively enhanced by decreasing SENP3 expression [18]. The depletion of SENP3 protects cells from ischaemia-induced cell death by prolonging the covalent binding of Drp1 and SUMO2/3, which suppresses the release of Drp1-mediated cytochrome c and caspase-involved cell apoptosis [18]. The discrepancy on the apoptosis effect of SUMOylated Drp1 is probably caused by the different experimental settings in these studies. It indicates that conjugation with a different SUMO for the same substrate protein may function specifically in pro- and anti-apoptosis under variable physio-pathological conditions.

The activation of SUMOylation is usually considered to protect cells against apoptosis in a wide range of cancer types, including lung cancer, breast cancer, colon cancer, prostate cancer, gastric cancer and brain tumour [19,20]. For instance, the silence of SUMO-related genes improves apoptosis of multiple cancer cells via acting on pluri-functional targets to affect multiple pathways, including P53-mediated apoptosis, NF-κB/Akt pathway and Wnt/β-catenin pathway [19,20]. The SUMO-mediated effects in cancers are probably different from the cardiovascular disease described above, which is dependent on the disease type, disease progression status, specific SUMO conjugation and SUMOylation level. In general, correcting the imbalance of SUMOylation and deSUMOylation is a new strategy for the treatment of certain diseases.

### 2.2. SUMO-mediated cell autophagy

Autophagy is a pro-survival mechanism induced by several cellular stress conditions, which cleans damaged cell components, responds to cytotoxic stimuli and resists lethal apoptosis [21]. Autophagy process includes autophagosome formation and maturation. When the inhibition of autophagy prevents cell death, it is referred to autophagic cell death. Among several PTMs upon with autophagy induction [21], SUMOylation is involved in macroautophagy/autophagy initiation [22,23].

**Figure 2.**
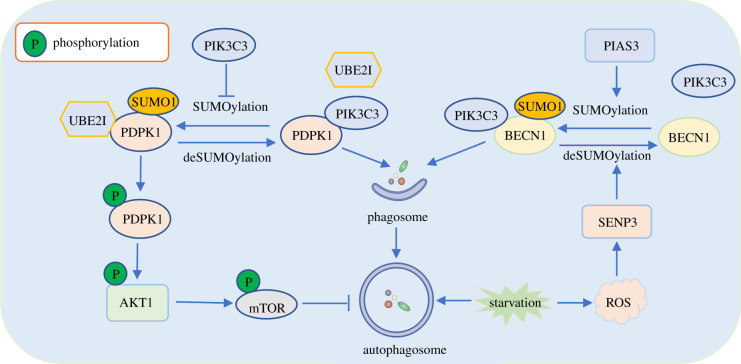
The same SUMO molecule is attached with two different target proteins PDPK1 and BCEN1, which exhibits an antagonistic role in regulating autophagosome formation. On the left side of the figure, SUMOylation of PDPK1 induces its phosphorylation to activate the AKT1-mTOR pathway and negatively regulate autophagy. In turn, the PIK3C3 negatively regulates PDPK1 SUMOylation by competing with SUMO E2 enzyme UBE2I for binding to PDPK1 during autophagy activation. Subsequently, the non-SUMOylation PDPK1 facilitates the expansion of the phagophore to form the enclosed autophagic vacuole. On the right side of the figure, BECN1 SUMOylation is potentiated by PIAS3 and subsequently facilitates the BECN1 interaction with the BECN1-PIK3C3 complex, enhancing autophagosome formation. Meanwhile, to avoid excessive autophagy, starvation can lead to the increase of ROS and the ROS-dependent accumulation of SENP3, which weakens the BECN1 SUMOylation to some extent.

The phosphatidylinositol 3-kinase catalytic subunit type 3 (PIK3C3) is necessary for macroautophagy/autophagy initiation [22,23]. PIK3C3 interacts with multiple SUMOylated target proteins, such as PDPK1 [22] and BECN1 to form several complexes to affect autophagy [23] (figure 2). Under physiological conditions, the SUMO1-conjugated PDPK1 occurs at K495 to induce its phosphorylation and facilitate the PDPK1-mediated activation of the AKT1-mTOR pathway, ultimately inhibiting autophagy (figure 2, left). However, when autophagy is activated, the SUMOylated PDPK1 is preferentially recruited and bound by PIK3C3, then PIK3C3 suppresses SUMOylation of PDPK1 by competing with SUMO E2 enzyme UBE2I for binding to PDPK1(figure 2, left). Subsequently, the non-SUMOylation PDPK1 stays in the ER pool via forming PDPK1-LC3 complex to enhance autophagosome formation [22]. BECN1, a mammalian homologue of yeast Vps30/Atg6, is SUMOylated by PIAS3 and deSUMOylated by SENP3. Upon cellular starvation, SUMO1-conjugated BECN1 is potentiated and subsequently facilitates the BECN1 interaction with the BECN1-PIK3C3 complex, enhancing autophagosome formation (figure 2, right) [23]. These examples suggest that the same SUMOylated substrate protein has a positive or negative regulation role in autophagy, which is dependent on the interacting partners with the substrate protein and the protein complex-regulated signal pathway.

SUMOylation relative autophagy is associated with multiple types of diseases. For instance, a high level of SUMO1 improves autophagic activation and accumulation of autophagic vacuoles, which promotes A*β* production and contributes to autophagic vacuoles-mediated Alzheimer disease pathogenesis [24]. On the contrary, the depletion of SUMO1 and UBC9 contributes to autophagic activation, induces autophagy-mediated breast cancer cell death and inhibits invasiveness of breast cancer cells [25]. So far, the suppression of abnormal SUMOylation level is a potential intervention strategy for cancer. SUMOylation contributes to maintain cardiac function and prevents from cardiac stress. The UBC9-mediated SUMOylation is sufficient to induce high levels of cardiac autophagy, which is associated with representing a novel strategy for ameliorating morbidity in proteotoxic cardiac disease [26]. These studies indicate a variable role for SUMOylation in different diseases, which illustrate the need for targeted therapies that remaining the SUMOylation activity of normal cells meanwhile reducing the activity of non-normal cells sufficiently with a SUMOylation inhibitor to prevent disease malignant transformation.

In addition, the SUMO pathway is regulated by autophagy [27]. UBC9 is physiologically degraded by autophagy upon with stress stimulation in cells. The stimulation of autophagic degradation by starvation or autophagy agonist, such as sorafenib treatment, induces UBC9 degradation, but the late autophagy inhibitors chloroquine and bafilomycin A1 suppress UBC9 degradation [27]. Thus, the relations between SUMOylation and autophagy are reciprocally interplaying. Dysregulated SUMOylation will lead to autophagy. Conversely, the autophagy phenotype has a profound effect on the balance of cellular SUMOylation level.

### 2.3. SUMOylation-regulated cell senescence

**Figure 3.**
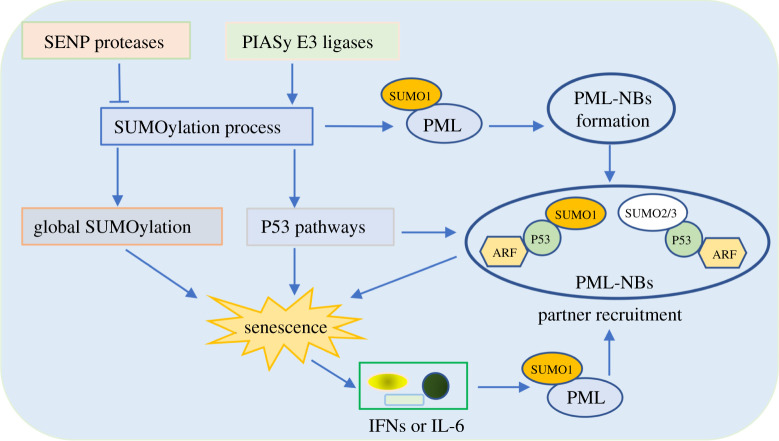
Crosstalk between SUMOylation and PML-induced cellular senescence. An increase of SUMOylation levels can stabilize the PML, which promotes PML-NB formation to induce senescence. In addition, SUMOylation enzymes regulate the SUMOylation process and mediate global SUMOylation, which affects PML-NB formation and partner recruitment of P53. Moreover, PML-NBs also potentiate P53 SUMOylation modification by recruiting ARF protein, ultimately facilitating the cellular senescence process. Conversely, senescent cells express specific cytokines (IFNs or IL-6) that enhance PML expression in a positive feedback loop.

Cellular senescence is the fundamental cellular fate of eukaryotes. Accumulating evidence indicates that SUMOylation is essential for governing senescence and ageing. The overexpression of SUMO2/3 enhances premature growth arrest of fibroblasts with features of oncogene-induced senescence [28]. Supporting this conclusion, in nucleus pulposus cells, SUMO2 gene silence improves protection against degradation and suppresses cell senescence [29]. Furthermore, the partial loss of SUMOylation relative enzyme activity or expression level, including SENP proteases and PIAS E3 ligases, also triggers PML and P53-related cellular defects (figure 3) [28–35].

Promyelocytic leukaemia nuclear body (PML-NB) contains three major components, PML, SUMO and UBC9, and functions as the main inducible factor of cellular senescence [32]. The spliceosome of PML is roughly divided into seven categories named PML I to PML VII, among which only PML IV isoform upregulation induces senescence in primary cells [33]. PML protein is the key factor driving the PML-NB formation. PML can be modified by SUMO on at least three Lys sites, including K65, K160 and K490, and SUMOylation of K160 is essential for PML ability [34,35]. Moreover, PML has also a SUMO interacting motif to recruit the key components of PML-NB allowing for a polymerization effect, ultimately leading to the formation of mature assemblies. In addition, PML has SUMO E3 ligase activity and mediates SUMOylation of PML-NB components such as P53 to trigger cell senescence. P53 is a key regulator of cellular senescence, and it conjugates to both SUMO1 and SUMO2/3 on the K386 residue under certain conditions [28,29,33]. P53 will be modified by SUMO1 under normal conditions, while P53 is attached by SUMO2/3 under oxidative stress conditions such as H_2_O_2_ treatment. The SUMOylation of P53 protein is detectable in PML-NB. And the overexpression of PML IV enhances P53-SUMO1 conjugation via interacting with ARF, which results in P53 stabilization and activation, ultimately triggering P53-driven senescence [33,35]. However, the SUMO2/3 modification of P53 requires the MDM2-ARF-L11 complex [33,35]. In the regulation of P53, SUMO2/3 clearly acts as a transcriptional modulator to activate or inhibit SUMO modification. This differential regulation paradigm exemplifies the object of SUMOylation to achieve substrate and isoform specificity. PML and senescence interplay reciprocally. PML overexpression leads to senescence. Conversely, senescent cells express specific cytokines (IFNs, IL-6) to enhance PML expression in a positive feedback way [35].

Preclinical studies have highlighted that the specific clearance of senescent cells in genetic animal models alleviates age-associated diseases including cardiovascular disease, fibrotic diseases, Alzheimer's disease and cancer that are closely associated with SUMOylation [36]. The acquired drug resistance of cancer cell is closely related to cell senescence. Genome unstable cancer cells will evade toxicity of anti-cancer treatments by acquiring a senescent-like phenotype (dormant state), and then bypass growth arrest, recovering the aggressive and uncontrolled proliferation [37]. SUMOylation alterations are also linked with multidrug resistance in cancer. Also, similar to unscheduled proliferative, oncogenic or DNA damage signalling, dysregulated SUMOylation can lead to senescence, with possible far-reaching effects on tumours and/or their microenvironment [38]. Hence, the intervention of certain drugs cause the imbalance of SUMOylation homeostasis in cancer cells, which ultimately triggers cells to fall into the dormant state of senescence and generates acquired resistance to such drugs. Taken together, targeting protein SUMOylation is a promising strategy for ageing resistance.

### 2.4. SUMOylation with other types of cell death

Pyroptosis is a newly discovered way of programmed inflammatory cell death, and it is regulated by SUMOylation [39]. Pyroptosis mainly mediates the activation of a variety of caspases including caspase-4, caspase-11 and caspase-5 via inflammasome, which leads to the release of pro-inflammatory mediators including cytokines, alarmins, IL-18 and IL-1β, ultimately resulting in cell destruction by means of pyroptosis. NLRP3 is the most widely characterized inflammasome in pyroptosis [39–41].

Under steady-state conditions, NLRP3 is SUMOylated by SUMO2/3 at multiple sites (K88, K133, K204, K552, K652 and K689) through SUMO E3 ligase MAPL, which leads to the inactivation of the NLRP3 inflammasome. On the contrary, upon the activation of NLRP3 inflammasome, the interaction between NLRP3 and MAPL is disrupted and NLRP3 is deSUMOylated by SENP6 and SENP7, which enhances caspase activation and IL-1β release to accelerate pyroptosis [39,40]. In addition, NLRP3 is SUMOylated with SUMO1 on residue Lys204 to facilitate inflammasome activation and IL-1β secretion. Conversely, the SUMOylation of NLRP3 is deSUMOylated by SENP3 [41]. Generally, the modification of SUMO1, SUMO2 or SUMO3 on the same protein may have an antagonistic effect on regulating substrate function.

Pyroptosis is not only limited to defensive effect but also can be related to the inflammatory cytokines that are released via the caspase-dependent pathway, which is related to the pathogenesis of rheumatoid arthritis [42]. Another study reveals that the expression level of SUMO1 and UBC9 is increased in an experimental collagen-induced arthritis model, and elevated SUMOylation promotes the progression of rheumatoid arthritis [43]. Thus, associations of SUMOylation, pyroptosis and rheumatoid arthritis need to be further explored for a comprehensive understanding of rheumatoid arthritis pathogenesis.

### 2.5. Possible association of SUMOylation with ferroptosis

Ferroptosis is a form of iron-dependent cell death driven by reactive oxygen species (ROS) from the Fenton reaction and subsequent lipid peroxidation, which is related to the SUMO pathway [44]. An elevated ROS concentration (and in particular H_2_O_2_) promotes lipid peroxidation by lipoxygenase or cytochrome P450 reductase, which is the key step in regulating ferroptosis [45]. Because of a ‘hyper metabolism’ phenotype, cancer cells generate more ROS than normal cells, and excessive ROS levels will induce cell death [46]. In multiple cancers, the SUMO pathway is hyperactive, which is thought to be a selective advantage to fight stresses. Hence one hypothesis is that this mechanism contributes to the greater tolerance of cancer cells towards elevated ROS and avoids oxidative stress-induced cell death [47]. In addition, several pathways, including a series of SUMO target proteins Keap1/Nrf2 [48,49], P53 [50], BECN1 [51] and NF2/YAP [52], are modified with susceptibility to ferroptosis. Among them, the Nrf2 protein functions as an antioxidant transcript factor that is closely related to ferroptosis inhibition [53], and it can be SUMOylated at the conserved site K110 [48,49]. Moreover, SUMOylation escalates the scavenging ROS activity of Nrf2, ultimately enhancing hepatocellular carcinoma cells to tolerate oxidative stress [48,49]. Numerous studies have also shown that scavenging ROS or enhancing antioxidant capacity effectively improves resistance to ferroptosis [54–56], further indicating that SUMOylation and ferroptosis are closely related.

Anti-oxidative enzyme glutathione peroxidase 4 (GPX4) is a central repressor of ferroptosis that suppresses peroxidation of membrane phospholipids, and it will be modified by SUMO to be involved in ferroptosis. The classical SUMOylation consensus motif sites including K74, K106 and K125 are the residues most likely to undergo SUMO modification according to bioinformatics analysis using three online software resources (SUMOplot, JASSA and GPS-SUMO) [2]. In addition, K125 residue of GPX4 locates in the GPX4 cationic area and ranks the highest possibility of the SUMOylated sites. GPX4 cationic area, encompassing R152, K125 and K135, interacts with phospholipid bilayer via the negative charges of phospholipids and then precisely directs the redox-active centre of GPX4 toward the hydroperoxide group in the fatty acid chain of the phospholipid, ultimately resulting in the suppression of membrane peroxide phospholipids [56]. Thus, GPX4 SUMOylation at K125 site may affect the interaction between GPX4 and membrane phospholipids to regulate ferroptosis, which is worthy of further verification.

## 3. Concluding remarks

The importance of SUMOylation has been established in multiple cellular death patterns. The fine balance of SUMOylation and deSUMOylation is the key modulatory factor for cellular death, ultimately contributing to the development of a spectrum of diseases. This has attracted considerable interest in drug discovery that targets members of the SUMOylation pathway. If such specificity can be realized, these chemical molecules may initiate cell death through several disparate pathways working in parallel, which would be highly effective cytotoxins. However, SUMOylation regulates certain cellular death patterns or substrate proteins positively or negatively depending on specific conditions, including SUMO isoforms, disease types and cell microenvironment. Because of this complexity of SUMOylation regulation, the treatment efficiency for SUMO-mediated diseases by increasing or decreasing SUMOylation level is determined by multiple factors. The small-molecule drug N106 is a SUMO activator, which was originally developed to treat heart failure [57]. Mechanistically, N106 significantly increases E1 activity and thereby increases SUMOylation of SERCA2, ultimately preventing heart failure. But long-term treatment may increase the risk of cancer or neurodegenerative diseases. Moreover, various natural or synthetic small-molecule inhibitors targeting SENPs, E1 enzyme and UBC9 have been described in the literature [58]. For instance, ginkgolic acid is an inhibitor of E1 enzyme [59], and 2D-08 is the UBC9-targeting inhibitor [60]. However, their inhibitory activity is generally in the high µM range with low specificity. The only highly specific and efficient inhibitor available to date is ML792 targeting SAE [58,61], which has recently been developed by Takeda Pharmaceuticals. This molecule has a promising anti-cancer activity in preclinical mouse models. However, ML-792 probably induces skin irritation and ulceration at the injection site in immunodeficient mice, suggesting that ML792 has some side effects with toxicity [61]. In addition, it is likely that cancer cells will develop resistance to ML792. Thus, this therapeutic strategy will require suitable dosing to maintain SUMOylation activity of normal cells unchanged and to reduce the activity of cancer cells sufficiently to prevent oncogene-driven malignant transformation. Another reasonable approach is to deliver an inhibitor of the SUMOylation machinery directly to cancer cells.

In conclusion, further characterization of the relevant mechanisms and consequences of protein SUMOylation is an important priority to intervene in dysregulation of SUMO modification. Thus, mapping the exact SUMOylation network with cellular death contributes to developing disease therapeutic strategies.

## Data accessibility

This article does not contain any additional data.
